# Interaction of 5-HTTLPR and SLE disease status on resting-state brain function

**DOI:** 10.1186/s13075-024-03276-y

**Published:** 2024-01-31

**Authors:** Lihua Ma, Yifan Yang, Shu Li, Bibhuti Upreti, Shuang Liu, Xiangyu Wang, Ru Bai, Yuqi Cheng, Jian Xu

**Affiliations:** 1 Department of Rheumatology and Immunology, First Affiliated Hospital of Kunming Medical University, Kunming, China; 2 Department of Psychiatry, First Affiliated Hospital of Kunming Medical University, Kunming, China

**Keywords:** Systemic lupus erythematosus, 5-HTTLPR, Resting-state functional magnetic resonance imaging

## Abstract

**Background:**

Neuropsychiatric involvement in systemic lupus erythematosus (SLE) is a common clinical manifestation. In SLE patients, cerebral function is a more sensitive predictor of central nervous system damage, and abnormalities in cerebral function may be apparent before substantial neuropsychiatric symptoms occur. The 5-hydroxynyptamine(5-HT) system has the ability to interact with the majority of the neurochemical systems in the central nervous system (CNS), influencing brain function. Serotonin transporter gene-linked polymorphic region (5-HTTLPR) is an essential element of the 5-HT system gene polymorphism and is directly related to the control of 5-hydroxytryptamine transporter (5-HTT)gene expression. The relationship between 5-HTTLPR and functional brain measurements in SLE patients requires more investigation because it is one of the most attractive imaging genetics targets for shedding light on the pathophysiology of neuropsychiatric lupus.

**Methods:**

Resting-state functional magnetic resonance imaging (rs-fMRI) images were collected from 51 SLE patients without obvious neuropsychiatric manifestations and 44 healthy volunteers. Regional homogeneity (ReHo), amplitude of low-frequency fluctuations (ALFF), and fractional amplitude of low-frequency fluctuations (fALFF) were selected as indicators for evaluating brain function. In accordance with the Anatomical Automatic Labeling template, the gray matter was divided into 116 regions. The mean ReHo value, mean ALFF value, and mean fALFF value of each brain region were extracted. 5-HTTLPR genotypes of all research objects were tested by polymerase chain reaction and agarose gel electrophoresis. Two-way analysis of covariance was used to investigate whether there is an interaction effect between SLE disease status and 5-HTTLPR genotype on resting-state brain function.

**Results:**

In SLE patients with S/S homozygosity, there were notably lower mean ReHo, mean ALFF, and mean fALFF values observed in the right parietal, inferior angular gyrus, and the right paracentral lobule compared to healthy controls. However, this distinction was not evident among carriers of the L allele. Within the S/S genotype, SLE patients exhibited decreased mean ReHo in the left posterior cingulate gyrus, reduced mean fALFF in the left caudate nucleus, and diminished mean ALFF in the left temporal pole: superior temporal gyrus, in contrast to the HC group. Conversely, no such differences were discerned among carriers of the L allele. Notably, among L allele carriers, SLE patients displayed a higher mean ReHo value in the right hippocampus compared to the HC group, while demonstrating a lower mean ALFF value in the left medial and paracingulate gyrus in contrast to the HC group. Conversely, these differences were not apparent among S/S homozygotes.

**Conclusions:**

Brain function in the right parietal and inferior angular gyrus and the right paracentral lobule is affected by the interaction effect of SLE disease status and 5-HTTLPR genotype.

## Background

Systemic lupus erythematosus (SLE) is an autoimmune disease that causes production of many autoantibodies due to aberrant immune system activation, culminating in the numerous organ damage [1]. One of the most typically damaged organs in SLE is the nervous system, and the accompanying central and peripheral nervous system dysfunction is known as neuropsychiatric systemic lupus erythematosus (NPSLE). According to published research, it affects between 12 and 95% of SLE patients [2]. The vast diversity of presentations of NPSLE, from typical headaches, cognitive abnormalities, and mood disorders to unusual manifestations such as Guillain–Barre syndrome and autonomic dysfunction, is one of the obstacles doctors frequently confront in diagnosing and managing patients with NPSLE [3]. In SLE patients, cerebral function is a more sensitive predictor of central nervous system damage, and abnormalities in cerebral function may be apparent before substantial neuropsychiatric symptoms occur.

Resting-state functional magnetic resonance imaging (rs-fMRI) is a noninvasive imaging technique that uses a blood oxygenation level-dependent (BOLD) signal to investigate brain function in a variety of central nervous system (CNS) diseases such as Alzheimer’s disease, Parkinson’s disease, depression, schizophrenia, and others [4–7]. Increasing amounts of study have used functional magnetic resonance imaging (fMRI)to investigate alterations in brain function in SLE patients and have revealed brain functional abnormalities even in non-NPSLE patients [8, 9]. rs-fMRI enables the reflection of brain function through multiple indicators. Primarily, it encompasses amplitude of low-frequency fluctuation (ALFF) and fractional amplitude of low-frequency fluctuations (fALFF), which effectively gauge the intensity of neuronal activity in specific localized brain regions [10]; ②Regional homogeneity (ReHo) is a measure of how well all neurons in a given area of the brain coordinate their spontaneous activity [11]. ReHo values were found to be significantly lower in non-NPSLE patients' fusiform gyrus and thalamus than in healthy controls, whereas they were higher in non-NPSLE patients’ parahippocampal and uncinate regions [9].

Despite studies demonstrating abnormal brain function in SLE patients, it is unclear what causes NPSLE. Thrombosis, autoantibodies, cytokines, and cell-mediated inflammation, as well as changes to the blood–brain barrier, are examples of potential pathogenic processes [2]. Additional research should be done on the effects of genetic variables, which are important in the pathophysiology of SLE, on the structure and function of the brain. Imaging genetics, a type of genetic association analysis that combines imaging and genetics, can investigate the impact of genetic variation on brain structure and function, as well as the relationship between neuropsychiatric disease risk genes, brain activity, clinical manifestations, and so on. One of the more common imaging genetics research targets is the 5-hydroxytryptamine (5-HT) pathway-related genes. As a neurotransmitter, 5-HT is a critical signaling molecule in the CNS, controlling nearly every part of the nervous system from the brain stem cell body. It indicates that the 5-HT system can interact with the majority of neurochemical systems in the CNS [12]. The 5-hydroxytryptamine transporter (5-HTT) is located on the presynaptic membrane of nerves and is responsible for regulating the levels of 5-HT by reabsorbing the 5-HT that has been released into the synaptic gap back to the presynaptic membrane. This mechanism establishes 5-HTT as a pivotal element in modulating synaptic transmission within 5-HTergic neurons. Its crucial role lies in the regulation of synaptic transmission within this neuronal network. Serotonin transporter gene-linked polymorphic region (5-HTTLPR) is an essential element of the 5-HT system gene polymorphism and is directly related to the control of 5-HTT gene expression. The L and S alleles of 5-HTTLPR are the most prevalent alleles. An imaging genetics research discovered that 5-HTTLPR L-allele carriers had substantially lower cortical volume in the right anterior midcingulate gyrus compared to S-allele homozygotes in a pooled sample of patients from the severe depressive disorder and healthy participants groups [13].

To the best of our knowledge, no studies have been conducted on the association between 5-HTTLPR and brain function indices in SLE patients. This work utilized rs-fMRI to evaluate the effect of 5-HTTLPR gene polymorphism and SLE disease status on resting-state brain function, providing preliminary information for SLE imaging genetics research.

## Materials and methods

### Participants

This study included SLE patients with no obvious neuropsychiatric manifestations who were admitted to the inpatient department of the Rheumatology and Immunology Department of the First Affiliated Hospital of Kunming Medical University. Therefore, the inclusion criteria were as follows: (1) the diagnosis of SLE is determined according to the Revised Criteria for the Classification of SLE developed by the American College of Rheumatology (ACR) in 1997 [14]; (2) being within the age range of 15 to 55 years old; (3) right handed; (4) voluntarily participating in the research and signing the informed consent.

The exclusion criteria were as follows: (1) patients with a history of head trauma; (2) patients with a history of drug or alcohol dependence; (3) patients suffering from other connective tissue diseases, blood system diseases, cardiovascular and cerebrovascular diseases, malignant tumors, etc.; (4) patients with parenchymal brain disease, CNS infection, epilepsy, and other neuropsychiatric diseases not caused by SLE; (5) patients who have contraindications to MRI (such as pacemaker, metal implants in the body, and claustrophobia); (6) pregnant or breastfeeding women; (7) those with abnormal brain structure indicated by conventional T1- and T2-weighted MRI; (8) the head movement is obvious during the scanning process, which may affect the results of MRI data preprocessing (in the head movement parameters, the translation > 2.0 mm, the rotation > 2.0°).

This study included healthy controls (HC) who were matched with the SLE group in terms of age, gender, and years of education.

### Testing for 5-HTTLPR

**Fig. 1:**
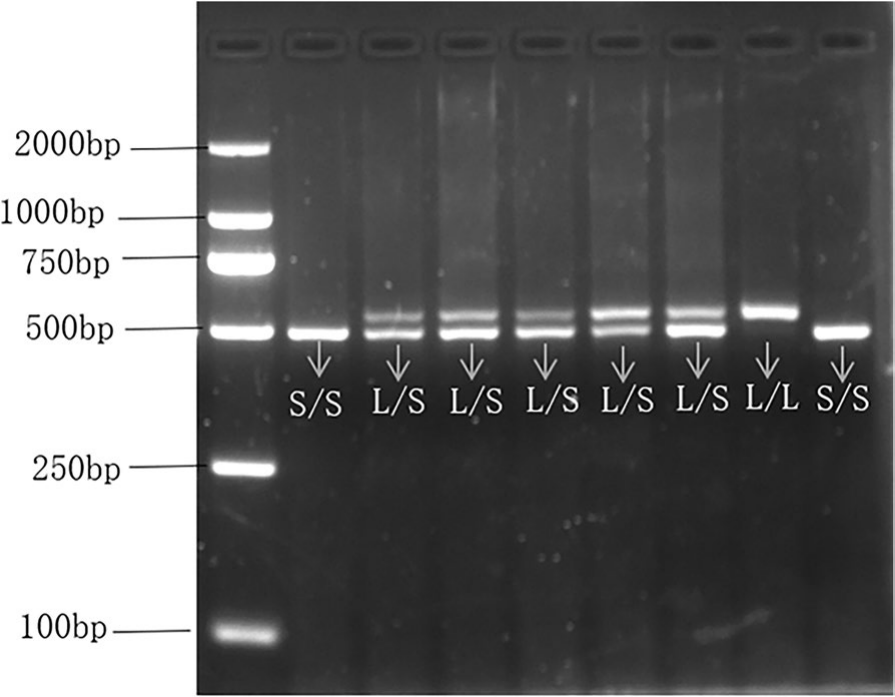
5-HTTLPR genotyping electropherogram. The identification of a sole band at 484 bp signified the S/S genotype, while a solitary band at 528 bp denoted the L/L genotype. The concurrent presence of bands at both 484 bp and 528 bp indicated the L/S genotype

(1) The DNA extraction process involved isolating DNA from 2 mL of peripheral venous blood, which was anticoagulated using 2% EDTA. This extraction was carried out following the guidelines provided by the TIANamp Blood DNA Kit DP348 from the supplier, Tiangen Biotechnology (Beijing) Co; (2) polymerase chain reaction (PCR) amplification of target genes: sense primer (5′-GGCGTTGCCGCTCTGAATGC-3′) and antisense primer (5′- GAGGGACTGAGCTGGACAACCAC-3′) were synthesized by Sangon Biotech Co., Ltd., Shanghai, China. 0.8 µl sense primer (10uM), 0.8 µl antisense primer (10 µM), 10 µl 2 × GoldStar Best MasterMix (Dye) (Jiangsu Kangwei Century Biotechnology Co., Ltd.), 2 µl template DNA and 6.4 µl double distilled water were mixed in one PCR tube. PCR was started with predenaturation at 95 °C for 10 min, followed by 10 cycles including denaturation at 94 °C for 30 s, annealing at 65 °C for 30 s (1 °C drop per cycle), and extension at 72 °C for 1 min. Then 30 cycles were performed including denaturation at 94 °C for 30 s, annealing at 64.3 °C for 30 s, and extension at 72 °C for 1 min. The last step was the final extension at 72 °C for 5 min. (3) Genotype reading: PCR amplified products were separated by electrophoresis in 3% agarose gel mixed nucleic acid dyes (voltage 140 V, 40 min). Bands of the target gene were visualized under an ultraviolet gel imaging system, Gel DocEQ (Bio-Rad Laboratories, Inc., CA, USA). PCR-produced products of the pair of primers used in this study had two lengths; 484 bp and 528 bp. The band with a length of 484 bp was identified as the S/S genotype, and the band with a length of 528 bp was identified as the L/L genotype. Bands with lengths of 484 bp and 528 bp appearing at the same time were identified as L/S genotype. The electropherogram of 5-HTTLPR genotyping is shown in Fig. 1.

### Rs-fMRI acquisition

The MRI scans for all subjects were conducted by a highly experienced radiologist, utilizing the same 1.5 T MRI scanner manufactured by General Electric (Twinspeed; GE Medical Systems, Milwaukee, WI, USA). Initially, plain scanning including routine T1-weighted image and T2-weighted image was conducted to rule out any intracranial organic lesions. An echo planner imaging sequence scan was used with the following parameters: repetition time = 2000 ms, echo time = 40 ms, thickness = 5 mm with an interslice gap of 1 mm, field of view = 240 mm × 240 mm, matrix size = 64 × 64, flip angle = 90°, number of excitation = 2.00, number of layers = 24, time point = 160. The total fMRI scan time was 320 s.

The rs-fMRI data was preprocessed on a DPARSF software based on the MatlabR2016a platform. As per the Anatomical Automatic Labeling template from MNI, the gray matter in each subject's brain was segmented into 116 regions. Among these, the initial 90 regions corresponded to the brain's gray matter, while the remaining 26 regions represented the gray matter of the cerebellum. The rs-fMRI data processing toolkit REST V1.8 was used to extract the mean ReHo value, mean ALFF value, and mean fALFF value of each region.

### Demographics and psychological assessment

The details of sex, age, weight, height, medical history, family medical background, personal history, and habitual hand use were recorded for both SLE patients and HC. Lupus disease activity was assessed using the SLE disease activity index-2000 version (SLEDAI-2000) [15]. We used Hamilton Depression Scale (HAMD) [16] and Hamilton Anxiety Scale (HAMA) [17] to assess levels of depression and anxiety in SLE patients. Scores on the two scales were recorded and evaluated by two systematically trained physicians and achieved good inter-examiner reliability after systematic training.

### Statistical analysis

Statistical software IBM SPSS Statistics 21 (IBM Inc. Armonk, NY, USA) was used for data analysis. Quantitative data following normal distribution were expressed as $\overline{x }$±s, and *t* test was used for between-group comparison; otherwise, the quantitative data of non-normal distribution data was expressed as *M* (*P*25%, *P*75%), and the Mann–Whitney *U* test was used for between-group comparison of two groups. The chi-square test was chosen when analyzing qualitative data. When *P* < 0.05, the difference was considered to be statistically significant.

The mean ReHo, mean ALFF, and mean fALFF values of each brain region were used as dependent variables, whereas diagnosis of lupus (SLE vs HC) and 5-HTTLPR genotype (L allele carriers vs S allele homozygous) were used as independent variables and age as a covariate for a two-way analysis of covariance to investigate an interaction between disease status and 5-HTTLPR genotype on resting-state brain function. If the strength of the interaction was statistically significant, post hoc analysis (Bonferroni method) was used to compare the differences between subgroups. When *P* < 0.05, the difference was considered to be statistically significant.

## Results

### Hardy–Weinberg genetic equilibrium test

According to the inclusion and exclusion criteria established in this study, a total of 51 SLE patients without obvert neuropsychiatric manifestations and 44 healthy controls were included. In order to test population representativeness of the two groups, Hardy–Weinberg genetic equilibrium test was used. It showed that both groups are in line with the Hardy–Weinberg genetic equilibrium law (Table 1), indicating that both samples are representative of the population.

**Table 1:** Hardy–Weinberg genetic equilibrium test

Group	5-HTTLPR genotype	Observed value	Expected value	*χ*^2^	*P*-value
SLE	L/L	3	4.12	0.60	0.74
	L/S	23	20.75		
	S/S	25	26.12		
HC	L/L	4	3.55	0.11	0.95
	L/S	17	17.90		
	S/S	23	22.55		

*SLE* Systemic lupus erythematosus, *HC* Healthy control

### General information, 5-HTTLPR genotype, and allele frequency

The gender distribution, age demographics, and educational levels within both the SLE and HC groups displayed no statistically significant variations (Table 2). In the SLE cohort, 26 individuals carried the L allele (3 L/L, 23 L/S), while 25 individuals were S/S homozygotes, resulting in L allele and S allele frequencies of 28.43% and 71.57%, respectively. Within the HC group, there were 21 L allele carriers (4 L/L, 17 L/S), alongside 23 S/S homozygotes, reflecting L allele and S allele frequencies of 28.41% and 71.59%, respectively. Notably, there were no statistically significant discrepancies observed in the 5-HTTLPR genotype or allele frequencies between the SLE and HC groups (Table 2). These findings indicate a congruence in the genetic backgrounds of the 5-HTTLPR gene in both cohorts, suggesting comparability in brain functionality.

**Table 2:** General information, 5-HTTLPR genotype and allele frequency

	SLE (*n* = 51)	HC (*n* = 44)	*χ*^2^/*T*	*P*-value
Gender [*n* (%)]			3.70	0.05^a^
Male	6 (11.80%)	12 (27.30%)		
Female	45 (88.20%)	32 (72.70%)		
Age (year)	29.76 ± 9.65	31.14 ± 8.06	0.63	0.53^b^
Education (year)	13.06 ± 3.39	13.25 ± 3.80	− 0.26	0.80^b^
5-HTTLPR genotype [*n* (%)]			0.10	0.75^a^
L/L + L/S	26 (51.00%)	21 (47.70%)		
S/S	25 (49.00%)	23 (52.30%)		
5-HTTLPR allele frequency			< 0.01	0.10^a^
L allele	29 (28.43%)	25 (28.41%)		
S allele	73 (71.57%)	63 (71.59%)		

*SLE*, systemic lupus erythematosus; *HC*, healthy control; *n*, number of cases

^a^2 × 2 chi-square test

^b^Independent sample *t*-test

### Clinical parameters in SLE patients with different 5-HTTLPR genotypes

SLE patients were grouped according to 5-HTTLPR genotype. Because the frequency of L/L genotype is very low, this study alloted L allele carriers (L/L + L/S) into one group. The general data such as gender, age, and clinical data such as SLE disease activity and mental scale were compared between the L allele carrier group and the S/S genotype group. No significant differences in gender, age, education level, SLEDAI, HAMA, and HAMD scores were seen between the two groups (Table 3).

**Table 3:** Clinical parameters in SLE patients with different 5-HTTLPR genotypes

	L/L + L/S (*n* = 26)	S/S (*n* = 25)	*χ*^2^/*T*	*P*-value
Female [*n*(%)]	22 (84.60%)	23 (92.00%)	0.15	0.70^a^
Age (year)	30.12 ± 10.36	29.40 ± 9.06	0.26	0.79^b^
Education (year)	13.04 ± 3.80	13.08 ± 2.97	− 0.04	0.97^b^
SLEDAI scores	9.85 ± 6.73	8.92 ± 7.02	0.48	0.63^b^
Disease activity (SLEDAI) [*n* (%)]			0.19	0.67^c^
None (0–4)	8 (30.80%)	9 (36.00%)		
Mild (5–9)	6 (23.10%)	6 (24.00%)		
Moderate (10–14)	5 (19.20%)	4 (16.00%)		
Severe (> 15)	7 (26.90%)	6 (24.00%)		
HAMD scores	8.38 ± 3.72	7.52 ± 4.84	0.72	0.48^b^
HAMA scores	6.46 ± 4.25	5.04 ± 4.15	1.21	0.23^b^
Depression/anxiety status [*n* (%)]				
Depression (≥ 7)	17 (65.40%)	12 (48.00%)	1.57	0.21^d^
Anxiety (≥ 7)	11 (64.70%)	6 (35.30%)	1.92	0.17^d^

*n*, number of cases

^a^Continuity correction chi-square test

^b^Independent samples *t*-test

^c^Kruskal-Wallis test

^d^2 × 2 chi-square test

### Gray matter function in SLE patients with different 5-HTTLPR genotypes

**Fig. 2:**
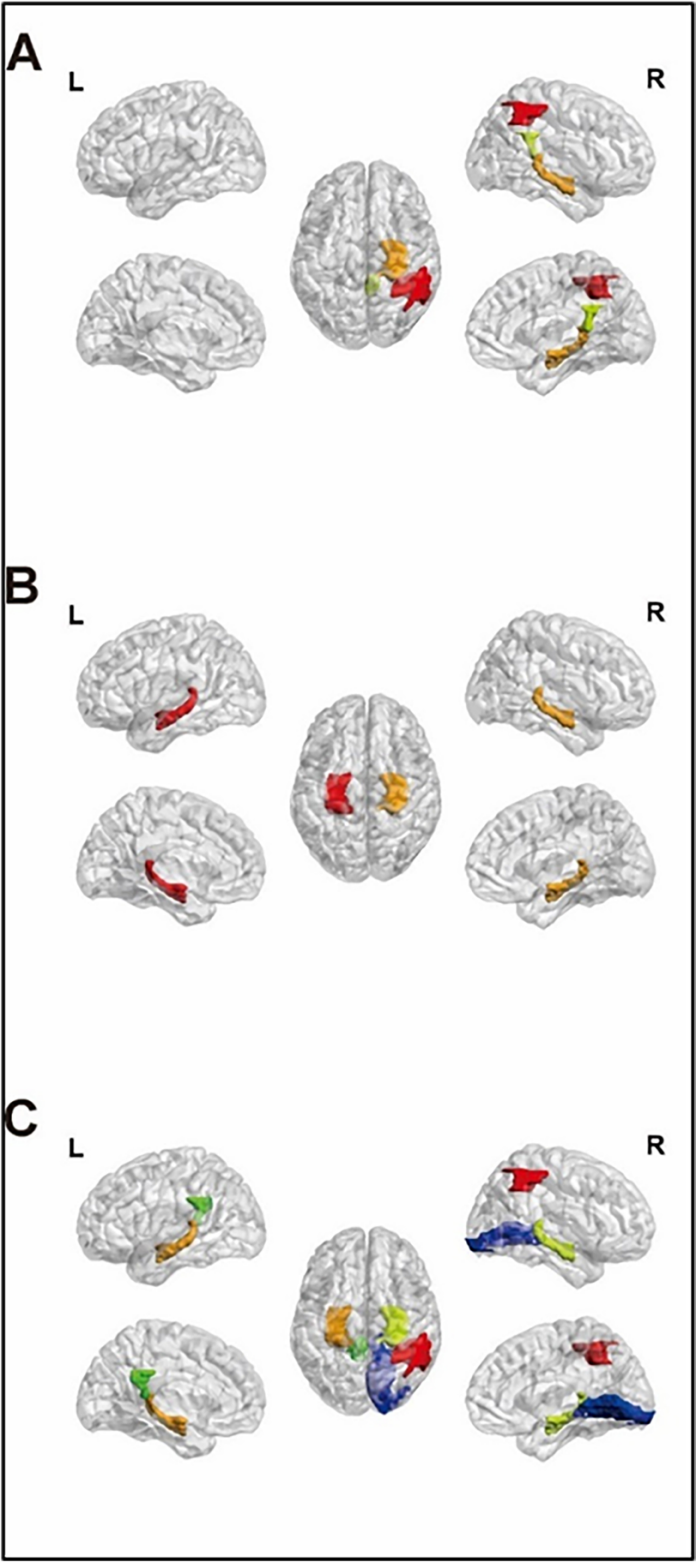
Gray matter function in SLE patients with different 5-HTTLPR genotypes. **A** ReHo, Red: Parietal_Inf_R, Orange: Hippocampus_R, Kelly: Cingulum_Post_R; **B** ALFF, Red: Hippocampus_L, Orange: Hippocampus_R; **C** fALFF, Orange: Hippocampus_L, Green: Cingulum_Post_L, Red: Parietal_Inf_R, Kelly: Hippocampus_R, Blue: Lingual_R; L: left; R: right

The mean ReHo value, mean ALFF value, and mean fALFF value of each gray matter area in the L allele carrier group and the S/S group in SLE patients were compared. The results showed that in SLE patients, the ReHo values of the right parietal and inferior angular gyrus, right hippocampus, and right posterior cingulate gyrus of the S/S group were lower than those of the L allele carriers; the ALFF values of the bilateral hippocampus were lower than those of the L allele carriers; the fALFF values of the right parietal and inferior angular gyrus, bilateral hippocampus, left posterior cingulate gyrus, and right lingual gyrus were lower than those of the L allele carriers (Table 4, Fig. 2).

**Table 4:** Gray matter function in SLE patients with different 5-HTTLPR genotypes

	AAL brain area	L/L + L/S (*n* = 26)	S/S (*n* = 25)	*T*/*Z*	*P*-value
ReHo	Parietal_Inf_R	0.93 ± 0.09	0.88 ± 0.08	2.35	0.02
	Hippocampus_R	0.99 ± 0.14	0.92 ± 0.08	2.36	0.02
	Cingulum_Post_R	1.07 ± 0.09	1.01 ± 0.10	2.07	0.04
ALFF	Hippocampus_L	1.05 (0.96,1.15)	0.98 (0.92,1.02)	− 2.13	0.03
	Hippocampus_R	1.00 (0.87,1.06)	0.92 (0.85,0.95)	− 2.13	0.03
fALFF	Parietal_Inf_R	0.96 ± 0.04	0.93 ± 0.03	2.87	0.01
	Hippocampus_L	0.98 (0.96,1.01)	0.95 (0.93,0.99)	− 2.62	0.01
	Hippocampus_R	0.99 (0.97,1.03)	0.96 (0.95,0.99)	− 2.86	< 0.01
	Cingulum_Post_L	0.99 ± 0.04	0.97 ± 0.04	2.07	0.04
	Lingual_R	0.96 ± 0.02	0.94 ± 0.02	2.33	0.02

*L* Left, *R* Right, *n* number of cases, *T* Test statistic for independent samples *t*-test, *Z* test statistic for Mann–Whitney *U* test

### Interaction between 5-HTTLPR gene and SLE disease state on brain function

**Fig. 3:**
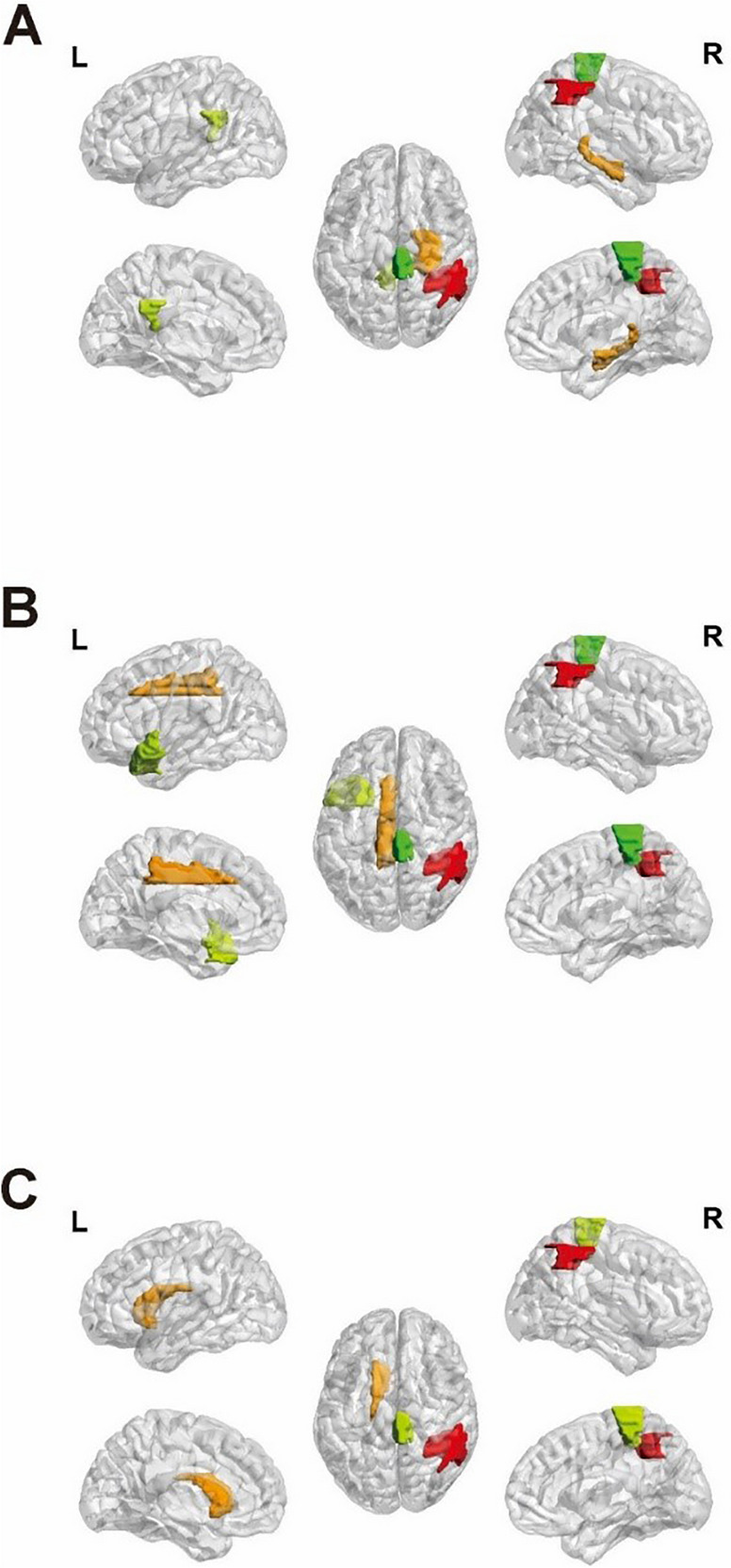
5-HTTLPR interacts with SLE disease state on gray matter function. **A** ReHo, Kelly: Cingulum_Post_L, Red: Parietal_Inf_R, Orange: Hippocampus_R, Green: Paracentral_Lobule_R; **B** ALFF, Orange: Cingulum_Mid_L, Kelly: Temporal_Pole_Sup_L, Red: Parietal_Inf_R, Green: Paracentral_Lobule_R; **C** fALFF, Orange: Caudate_L, Red: Parietal_Inf_R, Kelly: Paracentral_Lobule_R; L: left; R: right

The outcomes indicated that the mean ReHo, ALFF, and fALFF values within individual cerebellar gray matter regions remained unaffected by the interplay between SLE disease status and the 5-HTTLPR genotype. Among 90 Gy matter regions, the mean ReHo, mean ALFF, and mean fALFF values of the right parietal inferior angular gyrus and the right paracentral lobule were all affected by the interplay between SLE disease status and 5-HTTLPR genotype (Table 5, Fig. 3). Post hoc test analysis found that in the S/S genotype, the mean ReHo, mean ALFF, and mean fALFF values of the right parietal and inferior angular gyrus and right paracentral lobule of SLE patients were lower than those of the HC group, while such difference was not found among L allele carriers.

**Table 5:** Interaction between 5-HTTLPR gene and SLE disease state on brain function

	AAL brain area	Brain area number	*F* value	*P*-value
ReHo	Parietal_Inf_R	62	7.36	0.01
	Hippocampus_R	38	4.43	0.04
	Cingulum_Post_L	35	4.24	0.04
	Paracentral_Lobule_R	70	4.49	0.04
ALFF	Parietal_Inf_R	62	4.38	0.04
	Cingulum_Mid_L	33	4.47	0.04
	Temporal_Pole_Sup_L	83	10.73	< 0.01
	Paracentral_Lobule_R	70	5.54	0.02
fALFF	Parietal_Inf_R	62	8.38	< 0.01
	Caudate_L	71	4.46	0.04
	Paracentral_Lobule_R	70	4.43	0.04

*L* Left, *R* Right, *AAL*, Anatomical Automatic Labeling, *F-value*, test statistic for analysis of covariance

In addition, the mean ReHo value of the left posterior cingulate gyrus, the mean fALFF value of the left caudate nucleus, and the mean ALFF value of the left temporal pole: superior temporal gyrus were all affected by the interaction effect of SLE disease status and 5-HTTLPR genotype (Table 5, Fig. 3). Post hoc analysis found that in the S/S genotype, the mean ReHo value of the left posterior cingulate gyrus, the mean fALFF value of the left caudate nucleus, and the mean ALFF value of the left temporal pole: superior temporal gyrus of the SLE patients were lower than those of the HC group, while such difference was not found among L allele carriers. The mean ReHo values in the right hippocampus and the mean ALFF values in the left medial and paracingulate gyrus were all affected by the interaction between SLE disease status and the 5-HTTLPR genotype (Table 5, Fig. 3). Post hoc analysis found that among the L allele carriers, the mean ReHo value of the right hippocampus of SLE patients was higher than that of the HC group, and the mean ALFF value of the left medial and paracingulate gyrus was lower than that of the HC group, while such difference was not found among the S/S homozygotes.

## Discussion

There have been comparatively few studies investigating 5-HTTLPR in the context of SLE. In our study, there were no significant differences observed in the genotype and allele frequency of 5-HTTLPR between the SLE and HC groups. This aligns with the research findings reported by Li et al. [18]. This suggests that there isn't a direct association between genetic variations in 5-HTTLPR and susceptibility to SLE. Furthermore, our investigation found no correlation between genetic variations in 5-HTTLPR, SLE disease activity, depression, or anxiety. Xu et al. revealed that in their study, the average HAMD score among S/S homozygotes in SLE patients was higher than that among L allele carriers [19]. Additionally, within the SLE group, individuals experiencing depression exhibited a higher frequency of the S allele and S/S genotype compared to those without depression [19]. Despite these disparities with our study’s findings, the limited sample size in our investigation suggests the need for a re-evaluation of any conclusions. Confirmatory support through a larger follow-up study is essential.

The observed variance in gray matter functionality between L allele carriers and S/S homozygotes among SLE patients was primarily identified in the right inferior parietal angular gyrus and bilateral hippocampus. The S/S group exhibited poorer brain function indices across all affected brain areas compared to the L allele-carrying group. Our hypothesis attributes these findings to the variance in content and concentration of 5-HT within the synaptic cleft between S/S homozygotes and L allele carriers, given the S allele's association with reduced 5-HTT gene transcription and the L allele's association with increased 5-HTT gene transcription. The interaction between SLE illness severity and the 5-HTTLPR genotype resulted in alterations only in the resting-state brain function markers of specific gray matter brain regions. This interaction notably influenced the mean ReHo, mean ALFF, and mean fALFF values in the right parietal and inferior angular gyrus, along with the right paracentral lobule. This interaction effect highlighted that in individuals with the S/S genotype, the mean ReHo, mean ALFF, and mean fALFF values in the right parietal and inferior angular gyrus, as well as the right paracentral lobule, were lower among SLE patients compared to healthy controls. However, no such discrepancy was observed in L allele carriers. The functional roles of these regions are notable—the parieto-inferior angular gyrus primarily contributes to self-perception, executive functioning, and the integration of emotional and sensory information from facial stimuli [20]. On the other hand, the paracentral lobule governs motor and sensory innervation of the contralateral lower extremity [21], and it plays a role in motor performance, pain processing, and perception [22]. Abnormal paracentral lobular activity has been noted in individuals experiencing chronic pain, impacting sensory pain recognition or assessment [23]. Importantly, there is a lack of research exploring the relationship between the parietal and inferior angular gyrus, paracentral lobules, and 5-HTTLPR gene polymorphisms. This study highlighted the influence of the interplay between SLE disease state and 5-HTTLPR genotype on the three indices of brain function specifically in the right inferior parietal angular gyrus and the right paracentral lobule. It suggests that the 5-HTTLPR genotype modulates the impact of SLE disease on the brain function of these regions—specifically, individuals with the S/S genotype demonstrate a more pronounced effect of SLE disease on the brain function of these areas.

Moreover, the interplay between the SLE disease state and the 5-HTTLPR genotype influenced the mean ReHo value of the left posterior cingulate gyrus, the mean fALFF value of the left caudate nucleus, and the mean ALFF value of the left temporal pole: superior temporal gyrus. This interaction indicated that in individuals with the S/S genotype, the mean ReHo, fALFF, and ALFF values of these regions among SLE patients were lower compared to those of the HC group. Conversely, such differences were not observed among L allele carriers. Research has previously highlighted the significance of these brain regions. The posterior cingulate gyrus, known for its high metabolic activity in the resting state, is associated with self-evaluation [24], attention [25], formation of self-thoughts [26], and episodic memory, among other cognitive functions [27]. Similarly, the caudate nucleus, a component of the striatum, plays a crucial role in brain activity, particularly in cognitive function. Our findings indicate that the impact of SLE disease on brain function in the left posterior cingulate gyrus, left caudate nucleus, left temporal pole, and superior temporal gyrus was modulated by the 5-HTTLPR genotype. Specifically, individuals with the S/S genotype demonstrated a more pronounced effect of SLE disease on brain function in these regions.

Our investigation further demonstrated that the interaction between SLE disease status and the 5-HTTLPR genotype affected the mean ReHo values in the right hippocampus and the mean ALFF values in the left medial and paracingulate gyrus. This interaction implies that among SLE patients, those carrying the L allele exhibit heightened mean ReHo values in the right hippocampus and reduced mean ALFF values in the left medial and paracentral cingulate gyrus compared to healthy controls. Conversely, individuals with the S/S genotype do not display such distinctions. The limbic system, encompassing the hippocampus and cingulate gyrus, plays a pivotal role in emotion regulation and cognitive function. Notably, depressive, anxious, and cognitive symptoms are prevalent among SLE patients, potentially attributed to the functional impairment of adjacent brain regions caused by the disease. Our study highlighted that the 5-HTTLPR genotype of the subjects influenced the impact of SLE disease on the brain function indices of the hippocampus and cingulate gyrus. Specifically, individuals with the L/S or L/L genotype demonstrated a more pronounced effect of SLE disease on the brain function of these regions.

## Conclusions

In conclusion, our study sheds light on the intricate relationship between SLE, the 5-HTTLPR genotype, and brain function. By uncovering specific brain regions affected by this interaction, we highlight the potential influence of genetic variations on neurological manifestations in SLE patients. However, our findings are tempered by limitations such as sample size constraints, cross-sectional design, and the exclusive focus on the 5-HTTLPR genotype. Future research endeavors should encompass larger, longitudinal studies integrating diverse genetic and environmental factors. Addressing these limitations could provide a more comprehensive understanding of how genetic variations impact brain function in the context of SLE, ultimately guiding more targeted interventions and therapeutic approaches for affected individuals.

## Data Availability

The datasets generated and analyzed during the current study are not publicly available due to the protection of individuals’ privacy but are available from the corresponding author on reasonable request.

## Abbreviations

ALFF: Amplitude of low-frequency fluctuations
ACR: American College of Rheumatology
BOLD: Blood oxygenation level-dependent
CNS: Central nervous system
fALFF: Fractional amplitude of low-frequency fluctuations
fMRI: Functional magnetic resonance imaging
HC: Healthy controls
HAMD: Hamilton Depression Scale
HAMA: Hamilton Anxiety Scale
MRI: Magnetic resonance imaging
NPSLE: Neuropsychiatric lupus erythematosus
ReHo: Regional homogeneity
rs-fMRI: Resting-state functional magnetic resonance imaging
SLE: Systemic Lupus Erythematosus
SLEDAI-2000: SLE disease activity index-2000 version
5-HT: 5-Hydroxytryptamine
5-HTT: 5-Hydroxytryptamine transporter
5-HTTLPR: Serotonin transporter gene-linked polymorphic region
